# Treatment of Giant Cell Arteritis (GCA)

**DOI:** 10.3390/jcm11071799

**Published:** 2022-03-24

**Authors:** Alexis Régent, Luc Mouthon

**Affiliations:** 1 Service de Médecine Interne, Centre de Référence Maladies Auto-Immunes et Systémiques Rares d’Ile de France, APHP-CUP, Hôpital Cochin, F-75014 Paris, France; luc.mouthon@aphp.fr; 2 Institut Cochin, Université de Paris Cité, F-75014 Paris, France

**Keywords:** glucocorticoids, immunosuppressant, biologics, treatment, giant cell arteritis

## Abstract

Giant cell arteritis (GCA) is the most frequent primary large-vessel vasculitis in individuals older than 50. Glucocorticoids (GCs) are considered the cornerstone of treatment. GC therapy is usually tapered over months according to clinical symptoms and inflammatory marker levels. Considering the high rate of GC-related adverse events in these older individuals, immunosuppressive treatments and biologic agents have been proposed as add-on therapies. Methotrexate was considered an alternative option, but its clinical impact was limited. Other immunosuppressants failed to demonstrate a significant favourable benefit/risk ratio. The approval of tocilizumab, an anti-interleukin 6 (IL-6) receptor inhibitor brought significant improvement. Indeed, tocilizumab had a noticeable effect on cumulative GCs’ dose and relapse prevention. After the improvement in pathophysiological knowledge, other targeted therapies have been proposed, with anti-IL-12/23, anti-IL-17, anti-IL-1, anti-cytotoxic T-lymphocyte antigen 4, Janus kinase inhibitors or anti-granulocyte/macrophage colony stimulating factor therapies. These therapies are currently under evaluation. Interestingly, mavrilimumab, ustekinumab and, to a lesser extent, abatacept have shown promising results in phase 2 randomised controlled trials. Despite this recent progress, the value, specific condition and optimal application of each treatment remain undecided. In this review, we discuss the scientific rationale for each treatment and the therapeutic strategy.

## 1. Introduction

Giant cell arteritis (GCA) is the most frequent vasculitis. It typically affects large-sized arteries in people older than 50 years [1]. Ophthalmic, vertebral and external carotid-branch artery involvement is responsible for headaches and most of the ischemic symptoms described during GCA. Subclavian and axillary arteries as well as aorta involvement are now well described, owing to advances in imaging over the years [2]. Relapse rate differs during follow-up according to the initial symptom and/or vascular involvement [3,4]. These different clinical phenotypes might influence therapeutic strategies, although there is no agreement on the relevant examinations that should be performed at the time of diagnosis to identify large-vessel disease extension.

Glucocorticoids (GCs) have been the cornerstone of GCA medical treatment since the 1950s [5]. However, long-term use of GCs is responsible for diabetes, osteoporosis, infections, cardiovascular disease, behavioural effects or cognitive impairment [6]. Additionally, about 86% of patients experience GC-related side effects during follow-up [7,8]. Therefore, immunosuppressant (IS) therapy, including methotrexate (MTX) [9], has been used as an adjunct therapy despite the modest effect on vasculitis control. More recently, biologic agents and, specifically, tocilizumab (TCZ) have had a major effect on preventing relapse and as a GCs-sparing treatment [10]. Despite these alternatives, the optimal therapy for de novo or relapsing disease remains unclear, and recommendations vary among countries and over time [11].

In this review, we discuss the evidence-based data regarding available treatments or future therapeutic options and compare consensus guidelines regarding optimal treatment for GCA [11,12,13,14].

## 2. Glucocorticoids

GCs typically trigger a marked improvement within days [15]. Few studies have specifically focused on GCA initial therapy. In patients without stroke or transient or permanent vision loss, high-dose oral GCs therapy should be started as soon as possible to avoid stroke or visual impairment that may occur at the acute phase of the disease. Indeed, fast-tracked management of suspected GCA has been associated with an 88% reduction in permanent visual loss [16]. Another study confirmed the short-term benefit for sight, with a 2.1-fold higher relative risk of blindness in the conventional treatment group, with no change in long-term prognosis [17]. For uncomplicated disease, results for the use of initial intravenous (IV) GCs are conflicting [18,19]. In a randomised controlled trial (RCT) of 164 patients, 240 mg IV methylprednisolone had no significant long-term GCs-sparing effect [18]. In a smaller study, three methylprednisolone injections of 15 mg/kg/day allowed for a more rapid tapering of oral GCs [19]. In this study, 10 of 14 patients receiving IV GCs were taking <5 mg/day prednisone at 36 weeks as compared with 2 of 13 control patients (*p* = 0.003). Despite the increased rate of sustained remission, the small size of this RCT does not allow for definitive conclusions, and it is generally accepted that IV methylprednisolone therapy should be avoided in uncomplicated disease [12]. An initial starting dose has been proposed as a fixed dose [11,14,20] or weight-adjusted dose (0.5 or 0.7 mg/kg/day) [12,18], with few comparisons between GC induction schemes. Therefore, recommendations rely on expert opinion rather than strong evidence. Alternate day GC therapy increases the risk of relapse and should be avoided [21].

In patients with ischemic manifestations, methylprednisolone pulse therapy has been proposed to prevent further visual loss. Visual improvement was observed in 7% of 41 patients receiving IV methylprednisolone versus 5% of 43 patients receiving oral GCs (*p* = 0.672). In this study, early diagnosis and the immediate start of GC therapy were the keys to improvement [22]. The treatment of visual manifestations within the first day predicted improvement in a cohort of 69 patients with GCA and visual involvement [23]. Despite the lack of evidence for an additional effect of methylprednisolone pulse therapy, it is still the preferred option in recent recommendations for GCA with ischemic manifestations [11,14].

GC tapering schemes during GCA have not been extensively studied. Relapses tend to occur more often in patients receiving the fastest GC tapering protocol (40% at month 2 as compared to 20% in the slow tapering regimen group) [24]. Additionally, observational studies found that approximately 50% to 70% of patients achieve GC-free remission after a median follow-up of 2 to 3 years [7,8,25]. A more recent RCT found that 15% of patients on a 6-month GC tapering scheme could achieve GC-free sustained remission [10]. Thus, targeting a daily dose of prednisone of 20 mg/day at month 3, 10 mg/day at month 6 and 5 mg/day at month 12 seems too conservative. In addition, a delayed reduction in GC dose, defined as >10 mg above the target dose, was observed in nearly one-third of patients, and this might be responsible for a high burden of GC-induced side effects [6,26]. Relapses occurred when patients received 5 mg/day prednisone [25], and this low-dose GC therapy is often considered a security dose preventing further relapse.

## 3. Immunosuppressants

Despite improvement in the side effects prophylaxes, long-term use of GCs is associated with osteoporosis, infection or diabetes, which are a major concern in older individuals. Therefore, IS therapy has been evaluated for reducing patient exposure to GCs, relapse rate, and less frequently, remission induction rate (Table 1). Three RCTs evaluated MTX (7.5–20 mg/week) as an add-on therapy for newly diagnosed disease. These trials found no significant GCs-sparing effect [27] or reduction in relapse rate [28] despite limits (reduced dose of MTX in one trial [27] and alternate day GC therapy in another [28]). A third RCT found a reduced relapse rate (45% vs. 84.2%; *p* = 0.02) [29]. Following these conflicting results, an individual patient data analysis suggested hazard ratios for a first and second relapse of 0.65 (*p* = 0.04) and 0.49 (*p* = 0.02), respectively [9]. The real impact of MTX in real life is still debated [30], and a large multicentric RCT evaluating MTX versus TCZ (NCT03892785) is attempting to answer this delicate question. Findings from a small series of patients with relapsing GCA (*n* = 11) or polymyalgia rheumatica (*n* = 12) treated with leflunomide (LEF) showed a reduction in C-reactive protein level and GC daily dose [31]. Additionally, the results of a retrospective case series of 51 patients receiving LEF or MTX suggested a reduced duration of treatment achieved remission in patients receiving LEF [32]. However, the retrospective nature of the study, the limited size of the population and the rate of side effects must be considered.

In an RCT of relapsing GCA in clinical remission after GC therapy, 31 patients received azathioprine (AZA) as an add-on therapy or a placebo. After a 52-week follow-up, patients receiving AZA received a reduced dose of GCs (1.9 mg/day vs. 4.2 mg/day, *p* < 0.05) [33]. Despite the positive result, the modest clinical impact has not changed medical practice, and AZA is rarely used as an additional therapy. Dapsone was found to be effective in addition to GC as a first-line therapy or as a GCs-sparing agent in relapsing GCA [34]. However, patients experienced numerous side effects, including skin rash or agranulocytosis, and it is no longer considered a safe alternative [35]. Hydroxychloroquine was found to be associated with a high rate of skin toxic effects, together with increased relapse rate during follow-up, which prevented further evaluation [36].

Different retrospective studies highlighted that cyclophosphamide might be an option for refractory disease despite a high rate of side effects, ranging from 33% to 80%. A few patients received mycophenolate mofetil, and further studies are needed to better analyse the therapeutic potential of this drug for GCA [37]. An RCT evaluating the ability of cyclosporine A to induce remission and as a GCs-sparing agent gave negative results. A total of 26 of 29 patients in the cyclosporine A arm experienced side effects, so it should not be used for GCA [38].

## 4. Biologic Agents

Immunohistology studies have detected tumour necrosis factor α (TNF-α) in inflamed arteries. From this observation and case reports, several RCTs of TNF-α versus a placebo evaluated the clinical value of targeting TNF-α or its receptor (Table 2). Despite the strong rationale, all three RCTs had negative findings and failed to demonstrate a benefit for remission induction [39], GC therapy withdrawal [40], relapse rate, or as a GCs-sparing therapy [41]. The efficacy and safety of blocking T-cell activation with abatacept was evaluated in an RCT: 49 patients received abatacept on days 1, 15 and 29 and during week 8, then 41 patients who were in clinical remission were randomised to continue monthly abatacept or switch to a placebo [42]. Despite an increased relapse-free survival with abatacept (48% vs. 31% at week 52, *p* = 0.049), the phase 3 RCT was withdrawn (NCT03192969), although abatacept is still being evaluated versus placebo in another RCT for remission induction at year 1 (NCT04474847). In a study comparing TCZ, either IV or subcutaneous and abatacept subcutaneous, TCZ was found to more often induce vasculitis remission and to have a more important steroid sparing effect as compared to abatacept [43]. The weaker effect of abatacept might explain the phase 3 RCT withdrawal. Subcutaneous injections of ustekinumab at week 0, week 4 and then every 12 weeks was evaluated in 25 patients with refractory GCA [44]. In this cohort, the GC daily dose was reduced between baseline and week 52 and no patient experienced GCA relapse while under therapy, although five required a diminished interval between ustekinumab injections. In an open-label trial, ustekinumab was evaluated as an add-on therapy to treat active new-onset or relapsing disease [45]. The primary endpoint was the absence of relapse through week 52. Seven of 10 patients experienced relapse, and recruitment was stopped after the enrolment of 13 patients. The high rate of relapse might be due to the short and low-dose GC scheme used and may explain in part the disappointing results. A few patients with refractory GCA received anakinra, with seemingly good efficacy [46,47]. However, further data are required and this treatment cannot be recommended at this stage (ongoing trial: NCT02902731).

Since the early 1990s, interleukin 6 (IL-6) levels have been found to be associated with disease activity in GCA [48]. The potential interest of TCZ, a monoclonal antibody targeting the α-chain of the IL-6 receptor has emerged since 2010. After several case series [49], two RCTs confirmed that TCZ could have a major impact on relapse prevention or as a GCs-sparing agent [10,50]. Thirty patients with GCA were randomised 2:1 to receive IV TCZ monthly or placebo for 1 year: 85% of patients in the TCZ treatment arm were in remission at week 12 as compared with 40% in the placebo arm. Remission was maintained until the end of follow-up in the TCZ treatment arm, whereas only 20% of patients receiving the placebo were in remission at week 52 [50]. This striking improvement was confirmed and supported in the GIACTA study, a large phase 3 trial including 251 patients. More than half (56% and 53%) of patients receiving TCZ subcutaneously every week or every other week, respectively, showed sustained remission as compared with 18% and 14% of those receiving GCs only tapered over 26 or 52 weeks, respectively [10]. After this RCT, an open-label phase was continued, and the benefit of TCZ therapy remained significant for patients with new-onset or relapsing disease until the end of the 3-year follow-up [51]. Despite numerous relapses after TCZ withdrawal [49,52], the cumulative dose of GCs with TCZ treatment was considerably reduced. Safety concerns that might arise from a therapeutic strategy combining TCZ and GCs seem not to be justified in a selected population. A retrospective real-life study of 134 patients receiving TCZ only or combined with an IS agent, mainly MTX, suggested that treatment was safe and effective in unselected patients [53]. One-third of patients over 80 years old might experience mild to moderate TCZ-related side effects, which confirms the good safety profile of TCZ in the oldest patients [54]. In addition, for the first time, quality of life was improved with TCZ, as assessed by the Medical Outcomes Study 36-item Short Form Survey domain scores and was superior to the improvement observed in patients receiving GCs [55].

## 5. Future Perspectives

Following the progress in researching the pathophysiological pathways involved in GCA, several other molecules are currently being evaluated. Scarce reports suggest the potential interest of Janus kinase inhibitors [56]. In a proof-of-concept open-label study recruiting 15 patients, baricitinib 4 mg/day was found to be effective in patients with relapsing GCA. It allowed discontinuation of GC therapy in 13/14 patients at week 52 and was apparently well-tolerated despite the occurrence of adverse events in all but one patient [57]. The value of upadacitinib, a Janus kinase 1 and 1/3 inhibitor is being evaluated in a multinational RCT to induce sustained remission at week 52 (NCT03725202). Apart from the theoretical interest of secukinumab, an anti–IL-17 monoclonal antibody, a case report of a patient with both psoriatic arthritis and GCA suggested its clinical value [58]. Secukinumab is being evaluated versus a placebo in a phase 2 RCT added to a 26-week GC tapering scheme to enable sustained remission at week 28 (NCT03765788). Among 52 randomised patients, 70.1% of those receiving secukinumab reached the primary endpoint as compared to 20.3% in the placebo arm group (late abstract L19, ACR 2021). Another RCT will evaluate guselkumab, an anti–IL-23 monoclonal antibody, versus a placebo added to a 26-week GC tapering scheme to enable a sustained GC-free remission at week 28 (NCT04633447). Granulocyte-macrophage colony stimulating factor has emerged as a key cytokine playing a role in GCA inflammatory vascular lesions. A phase 2 RCT versus a placebo showed that the subcutaneous injection of 150 mg of mavrilimumab every other week could increase the time to relapse at week 26. In total, 83.2% of patients receiving mavrilimumab and 49.9% of those receiving placebo showed sustained remission at week 26 (late abstract L06, ACR2020). Sarilumab, another IL-6 receptor inhibitor, was evaluated in a large phase 3 RCT (NCT03600805). Unfortunately, the COVID-19 pandemic outbreak led to early discontinuation of the trial after the enrolment of 83 patients. Preliminary results available at ClinicalTrials.gov suggest the efficacy of sarilumab, although the results should be interpreted with caution. Apart from neutropenia, no safety concern was identified.

## 6. Therapeutic Strategy and International Recommendations

**Figure 1:**
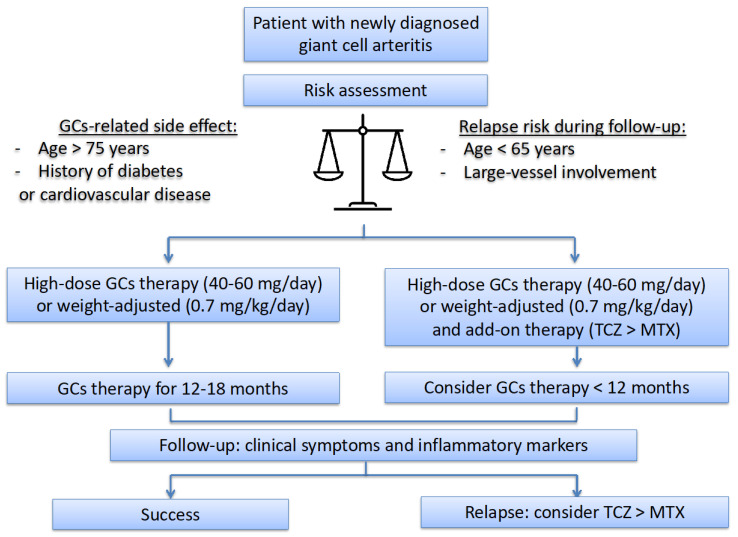
Proposed algorithm for treating giant cell arteritis. GCs: glucocorticoids; MTX: methotrexate; TCZ: tocilizumab.

Treatment with high-dose GC therapy allows for clinical and biological remission in patients with GCA. There are no evidence-based data to support the use of a higher initial dose of GCs for patients with specific manifestations. The improvement in visual manifestations seems related to the delay between symptoms and treatment rather than the GCs’ dose. In this context, high-dose IV methylprednisolone is considered the best treatment for patients with visual manifestations, on an expert opinion basis [11,12,13,14]. Unfortunately, only scarce findings can help identify patients who will experience relapse or will have GC-related side effects during follow-up. Therefore, combining GCs with an IS or biologic agent at the time of diagnosis is guided by the estimated risk associated with GC treatment. This is the ground-based approach proposed by European experts [11,13] and our team (Figure 1). It differs from the most recent recommendations established by US practitioners who advise a GCs-sparing agent in most patients and favour TCZ over MTX and other IS agents [14]. The youngest patients and those with large-vessel involvement may frequently experience relapse [4,8,59,60]. These patients who have clinical features overlapping with Takayasu arteritis may have some benefit from the initial prescription of GCs-sparing therapy at the time of diagnosis. Moreover, individuals > 75 years old and those with cardiovascular disease or diabetes might have more side effects from GC therapy [8]. The disease-related specific manifestations and medical history tend to help identify patients who require GCs-sparing therapy at the time of diagnosis (Figure 1).

Previously, treatment was considered to last 18 to 24 months [12]; the recommended length was progressively decreased from 12 to 18 months [13] to an individually tailored protocol based on clinical symptoms and biological results [14]. This reduced length of treatment significantly differed from the European recommendations, advising against a rapid tapering regimen [11]. It is probably based on the indication of biologic and IS agents, which are considered the first-line therapy for most patients in the US guidelines [14] but is considered a rescue therapy for most patients in the European ones [11,12,13]. Of note, treatment length > 2 years and relapse is associated with GC-related side effects, which should prevent the undue use of GCs for treating only relapse [61,62]. Sight threatening relapses are extremely rare, whereas-GC-related adverse events occur in most patients. We therefore recommend a 12–18 month treatment with GCs, and a reduced length may be considered in frailer patients.

Follow-up is based on regular clinical and biological evaluation. An increase in levels of inflammatory markers without any clinical signs suggestive of disease activity should not be considered a relapse and requires additional investigations, including searching for infection [14]. A consensus-based distinction between a major relapse defined by relapse with ischemic manifestations and minor relapse emerged in the European League Against Rheumatism recommendations [11]. Major and minor relapses differ in the proposed dose of GCs that should be used in association with IS or biologic agents: 40 to 60 mg/day for major relapses and the previously effective GCs’ dose for minor relapses. A retrospective study found that jaw claudication was the ischemic event in 44% of major relapses [63], which questions the implication in everyday patient care. Indeed, no additional data have been published to support that ocular manifestations, aortitis and limb ischemia are less responsive to standard therapy in patients with relapse. In contrast, vascular manifestations are well associated with further relapse during subsequent follow-up and should encourage the prescription of an IS or biologic agent in these patients. Comparing GCA and rheumatoid arthritis management, some questions still need to be answered. Is there a benefit to introducing IS or biological therapy early in GCA course in terms of sustained remission induction or time to relapse [64]? In patients receiving GC add-on therapy, do we have to consider an increased administration interval and slow tapering of IS or biological therapy to prevent further relapses [65]?

## 7. Non-Immunosuppressive Therapy

The increased short-term risk of ischaemic events raises the question of vascular event prevention. Despite several retrospective studies, whether low-dose aspirin could confer a significant protection is unclear. Indeed, two retrospective studies demonstrated a significant decrease in vascular ischemic events in patients receiving antiplatelet therapy [66,67]. A meta-analysis suggested that low-dose aspirin may have a marginal benefit when combined with GCs at the initial phase of the disease [68]. Disregarding these data, recommendations have advised following international guidelines for vascular disease prevention without any specificity for individuals with GCA [11,13,14]. The value of anticoagulants is not well documented. Different studies evaluated statins as a GCs-sparing agent but did not find any clinical benefit [69,70].

Despite the increasing use of IS or biologic agents during GCA, there is no specific concern regarding treatment in this frail population following the COVID-19 pandemic. The main risk for severe SARS-CoV-2 infection arises from the age of patients and associated comorbidities. Unlike rituximab in patients with anti-neutrophil cytoplasm antibody-associated vasculitis, treatment with GCs, MTX or TCZ modestly increased COVID-19 severity [71]. The vaccination response might be impaired, and treatment risks should be carefully balanced with benefits from GC add-on treatments. Finally, polymyalgia rheumatica or GCA might develop soon after vaccination [72]. However, a fortuitous occurrence must be considered.

## 8. Conclusions

Major efforts have been made recently to develop GC-reduced therapeutic strategies in GCA. This work questions the value of GCs in the disease. Is it the feared treatment to avoid in this frail population? We acknowledge that a short GC therapy has a rapid effect on vasculitis control. Better defining which patients might benefit from alternative therapy is crucial to appropriately using each treatment within a safe and cost-effective strategy. TCZ has the strongest evidence as a GC add-on therapy in patients with newly diagnosed or relapsing GCA. In the future, head-to-head comparisons of biological therapies might be required to build an efficient therapeutic strategy in these patients. Available GC add-on therapies have a steroid sparing effect and reduce relapse occurrence but fail to cure GCA, warranting further research.

## Figures and Tables

**Table 1:** Results of randomised controlled trials evaluating immunosuppressants for giant cell arteritis (GCA).

Author, Year	Number of Patients	Disease Activity	Drug Evaluated	GC Therapy	Main Primary Outcome	Result
Azathioprine						
De Silva et al., 1986	31	GCA in remission	Azathioprine 150 mg/day	GCs ≥ 5 mg/day Tapering 1 mg/month	Not specified (studied GCs daily dose)	Positive at month 12
Dapsone						
Liozon et al., 1993	48	New-onset GCA	Dapsone 50–100 mg/day	0.7–1.0 mg/kg/day Tapering ≈ 14 months	Relapses	Positive but dapsone-related side effects
Hydroxychloroquine						
Sailler et al., 2009	74	New-onset GCA	Hydroxychloroquine 400 mg/day	0.7 mg/kg/day Tapering ≈ 16 months	Remission > 3 month at the end of follow-up	Negative and hydroxychloroquine-related side effects
Methotrexate						
Spiera et al., 2001	21	New-onset GCA	MTX 7.5–20 mg/week	>40 mg/day. Suggested tapering ≈ 4 months	Cumulative dose of GCs at year 2	Negative
Jover et al., 2001	42	New-onset GCA	MTX 10 mg/week	60 mg/day. Tapering ≈ 6 months	Cumulative dose of GCs and relapses	Positive
Hoffman et al., 2002	98	New-onset GCA	MTX 0.15–0.25 mg/kg/week; max 15 mg/week	1 mg/kg/day and <60 mg/day; alternate day tapering ≈ 6 months	Relapses	Negative
Cyclosporine A						
Schaufelberger et al., 2006	60	New-onset GCA	CsA 2 mg/kg/day reduced or increased up to 3.5 mg/kg/day	Not specified	Cumulative dose of GCs and relapses	Negative. Numerous side effects

CsA: cyclosporin A; GCs: glucocorticoids; MTX: methotrexate.

**Table 2:** Results of randomised controlled trials evaluating biologics during GCA.

Author, Year	Number of Patients	Disease Activity	Drug Evaluated	GC Therapy	Main Primary Outcome	Result
Anti-TNF therapy						
Hoffman et al., 2007	44	New-onset GCA	Infliximab 5 mg/kg W0, 2 and 6 and every 8 weeks	Tapering < 6 months	Relapses at W22	Negative
Martinez-Taboadda et al., 2008	17	GCA in remission under GC > 10 mg/day. GC-related side effects	Etanercept 25 mg × 2/week	Tapering < 4 months (depending on initial daily dose)	GC-free remission at M12	Negative
Seror et al., 2013	70	New-onset GCA	Adalimumab 40 mg at W2, 4, 6, 8, 10	0.7 mg/kg. Tapering ≈ 10 months	Remission at W26 with GC < 0.1 mg/kg	Negative
Abatacept (CTLA4–Ig)						
Langford et al., 2017	49	New-onset or relapsing GCA Abatacept 10 mg/kg D1, 15, 29, 56 and GCs 40–60 mg/day for remission induction	41 patients randomised at W12, abatacept 10 mg/kg/4 weeks	20 mg/day at randomisation. Tapering until W28	Relapse-free survival	Positive
Tocilizumab (IL-6 receptor inhibitor)						
Villiger et al., 2016	30	New-onset or relapsing	Tocilizumab 8 mg/kg/month	1 mg/kg/day Tapering ≈ 9 months	Remission at W12 with GCs 0.1 mg/kg. Normal ESR and CRP	Positive
Stone et al., 2017	251	New-onset or relapsing	Tocilizumab 162 mg/week or 162 mg every other week	20–60 mg/day. Tapering 26 or 52 weeks	Prednisone-free remission at W52	Positive
Mavrilimumab (GM-CSF receptor-α inhibitor)						
Cid et al., 2020	70	New-onset or relapsing	Mavrilimumab 150 mg every other week	20–60 mg/day. Tapering 26 weeks	Relapse at W26	Positive
Secukinumab (IL-17A inhibitor)						
Venhoff et al., 2021	52	New-onset or relapsing	Secukinumab 300 mg/week (5 doses) then 300 mg/4 week until W48	25–60 mg/day. Tapering 26 weeks	Sustained remission at W28	Positive

CRP: C-reactive protein; CTLA4: cytotoxic T-lymphocyte-associated protein 4; D: day; ESR: erythrocyte sedimentation rate; GCs: glucocorticoids; GCA: giant cell arteritis; GM-CSF: Granulocyte-macrophage colony stimulating factor; Ig: immunoglobulin; IL: interleukin; M: month; TNF: tumor necrosis factor; W: week.

## Abbreviations

AZA	azathioprine
GCs	glucocorticoids
GCA	giant cell arteritis
IL	interleukin
IS	immunosuppressant
IV	intravenous
LEF	leflunomide
MTX	methotrexate
RCT	randomised controlled trial
TCZ	tocilizumab
TNF	tumor necrosis factor
